# Day/night variations of myeloid and lymphoid cell subsets in the murine inguinal lymph node

**DOI:** 10.1002/2211-5463.70137

**Published:** 2025-11-05

**Authors:** Paula M. Wagner, César G. Prucca, Lucia Boffelli, Virginia A. Piqueras, Silvia G. Correa, Mariana Maccioni, Mario E. Guido

**Affiliations:** ^1^ CIQUIBIC – CONICET, Departamento de Química Biológica Ranwel Caputto, Facultad de Ciencias Químicas Universidad Nacional de Córdoba Córdoba Argentina; ^2^ CIBICI – CONICET, Departamento de Bioquímica Clínica, Facultad de Ciencias Químicas Universidad Nacional de Córdoba Córdoba Argentina

**Keywords:** adaptive immunity, biological clock, circadian rhythms, innate immunity, lymphoid and myeloid cells

## Abstract

Circadian rhythms orchestrate physiological processes, including immune function, across a 24‐h cycle. This study investigates the temporal distribution of immune cell populations in healthy mice entrained to a 12:12‐h light–dark cycle. Inguinal lymph node (iLN) samples were collected at zeitgeber times (ZT) 0 (lights on) and ZT12 (lights off) to assess immune cell composition. A significantly higher proportion of natural killer T (NKT) cells and neutrophils was observed at ZT0 compared to ZT12, while dendritic cells, macrophages, and natural killer (NK) cells showed no significant temporal variation. Additionally, adaptive immune cells, particularly Programmed cell Death protein 1^+^ (PD1^+^) Cluster of differentiation 4^+^ (CD4^+^) and PD1^+^ Cluster of differentiation 8^+^ (CD8^+^) T cells, were more abundant during the light phase. These findings suggest a diurnal pattern in immune readiness, with implications for optimizing immunotherapeutic interventions based on circadian timing.

## Graphical abstract

The circadian system is involved in the temporal regulation of the immune system. Our study reveals that two innate immune populations, NKT cells and neutrophils, predominate at the beginning of the day in healthy mice, highlighting how the time of day influences immune responses. These results could have implications for optimizing immunotherapeutic strategies in cancer treatment. Image adapted from Servier Medical Art (https://smart.servier.com/), licensed under CC BY 4.0 (https://creativecommons.org/licenses/by/4.0/)

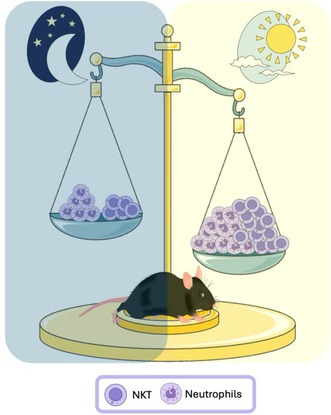

**Abbreviations**
CD4: Cluster of differentiation 4
CD8: Cluster of differentiation 8
CICUAL: Institutional Committee for the Care and Use of Experimental Animals
Cry: cryptochrome
FBS: fetal bovine serum
FDA: Food and Drug Administration
iLN: Inguinal lymph node
LD: light/dark
NK: natural killer
NKR: natural killer receptor
NKT: natural killer T
PBS: phosphate‐buffered saline
PD1: Programmed cell death protein 1
Per: period
ZT: zeitgeber time

Circadian rhythms are adaptive mechanisms that enable living organisms to synchronize with the Earth's rotational period, providing an evolutionary advantage in anticipating and adapting to a changing environment [1]. These rhythms are endogenous and self‐maintained oscillations with a periodicity of ∼24 h. Feeding and temperature cycles also adjust the circadian clock's timing to maintain homeostasis and physiology. At the molecular level, circadian machinery involves a set of clock genes and proteins interacting in transcriptional, translational, and post‐transcriptional feedback loops. In mammals, the positive elements CLOCK and BMAL1 activate the expression of the period (*Per*) and cryptochrome (*Cry*) genes, whose protein products inhibit the CLOCK:BMAL1 transcriptional activity. The heterodimer CLOCK:BMAL1 also activates the expression of clock‐controlled genes involved in a *plethora* of cellular and physiological functions (reviewed in [2, 3]). The circadian clock regulates numerous biological functions, including innate and adaptive immune responses, demonstrating significant daily rhythms in rodents and humans. These rhythms are evident in oscillations of immune cell counts, lymphocyte proliferation, and cytokine levels, underscoring the importance of timing in immune system effectiveness [4, 5, 6, 7, 8]. Moreover, monocytes [9, 10], macrophages [11, 12], neutrophils [13, 14], dendritic cells [15], and lymphocytes [15, 16] also show circadian variations in the expression of clock genes, which strongly suggests a functional clock machinery in immune cells. In addition, a strong circadian oscillation in circulating neutrophil numbers has been reported, resulting from a highly regulated process of release and clearance (reviewed in [17]). During these processes, the chemokine CXCL12 and its receptor CXCR4 represent a key retention signal for neutrophils in the bone marrow [18, 19]. On the contrary, signals from the sympathetic nervous system and the circadian regulation of CXCL12, among others, promote the return of these cells to the bone marrow between ZT5 during the day and ZT13 at night in mice [20]. Circadian regulation was also observed in the infiltration of neutrophils into *naïve* tissues, with a peak denoted at night [21].

As different researchers have evidenced, the host organism possesses a functional clock that operates in most of its cells, tissues, and organs, even in cancer cells, which impacts tumor development, progression, and metastasis (reviewed in [22, 23]). It was also demonstrated that susceptibility to infection [24], rheumatoid arthritis symptomatology [25], or asthma [26], as well as parameters in clinical diagnosis and pharmacological response [5] were shown to be time‐of‐day dependent. Based on the evidence, it becomes clear that the immune system operates as an exact and time‐regulated network governing the physiology of the entire organism in terms of both health and disease. As a vigilant guardian, it protects against infections and defends the body from tumor cells [8].

In line with this, we previously demonstrated that the circadian clock significantly influences tumor progression. We observed a marked increase in tumor growth when tumor cells were injected at night compared to injections made at the beginning of the day [27]. In addition, we found that Bortezomib—a well‐established proteasome inhibitor approved by the federal drug administration (FDA) for use in multiple myeloma—administered at night in tumor‐bearing mice significantly improved the chemotherapy response when compared to mice treated at the start of the day.

This insight highlights the potential for optimizing cancer therapies by considering the medication and when it is given [27]. Based on the above‐described phenomena, in this study, we aimed to examine the immunological cell populations present in draining lymph nodes in basal conditions (non‐tumor‐bearing mice) at the beginning of the day (ZT0) or night (ZT12).

## Materials and methods

### Mice

Wild‐type C57BL/6 mice (8–12 weeks of age) were purchased from BIOPROAL‐CONICET UNC (Córdoba, Argentina). Animals were housed under a regular 12‐h light/dark (LD) cycle (lights on at 7:00 AM and lights off at 7:00 PM) with food and water *ad libitum*. Time is expressed as “Zeitgeber Time.” Mice were euthanized by cervical dislocation, in accordance with institutional animal care guidelines, at ZT0 and ZT12 when lights were turned on and off, respectively. All animal procedures followed a protocol approved by the local CICUAL (Institutional Committee for the Care and Use of Experimental Animals), ethics approval number RD‐2022‐717‐E‐UNC‐DEC#FCQ.

### Isolation of inguinal lymph node mononuclear cells

iLN mononuclear cells were obtained by mechanical disintegration through metal meshes using a 6‐well plate as a reservoir and collected in 1× PBS (phosphate‐buffered saline) ‐ 2% fetal bovine serum (FBS).

### Surface staining and flow cytometry

Surface staining of single‐cell suspensions of draining lymph nodes was performed using standard protocols [28, 29]. Cells were counted using trypan blue exclusion in a Neubauer hemocytometer to assess cell concentration and viability. For immune cell characterization by flow cytometry, 1*10^6^ cells per sample were stained with a Live/Dead fixable cell stain kit (LIVE/DEAD Fixable Aqua Dead Cell Stain—Thermo Fisher Scientific, Waltham, MA, USA) to exclude dead cells in all experiments. Surface staining was performed in FACS buffer (1× PBS, 1 mm EDTA, 25 mm HEPES pH 7.0, 1% FBS) with the following antibodies: anti‐CD45, anti‐CD3, anti‐CD4, anti‐CD8, anti‐CD11b, anti‐LY6G, anti‐NK1.1, and anti‐Programmed cell death protein 1 (anti‐PD1) at room temperature for 30 min. Detailed antibody information can be found in Table S1. Flow cytometry was performed using an LSR Fortessa cytometer, and data were analyzed using flowjo software.

### Statistical analysis

The percentage of cells in each population was normalized by factorization to allow comparison across different experiments before performing the statistical analysis. Statistical analyses involve unpaired t‐tests to evaluate the time effects or Mann–Whitney test when the normality of residuals was infringed. In all cases, significance was considered at *P* < 0.05. All statistical analyses were performed with Prism 8.0.2 graphpad Software.

## Results

**Fig. 1:**
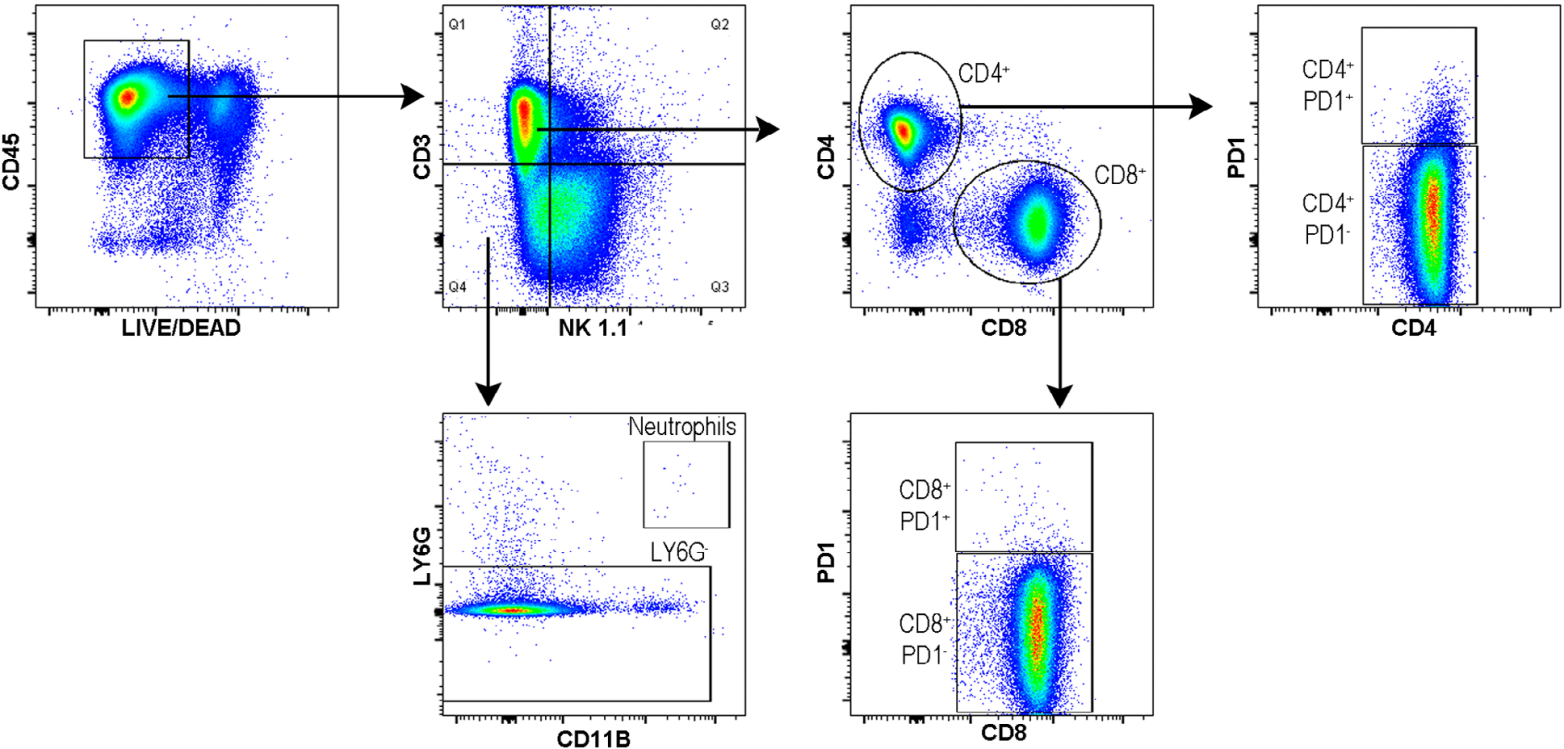
Schematic representation of the gating strategy used to analyze the different immune populations presented herein.

We previously demonstrated that tumor growth and chemotherapeutic treatment are time‐of‐day‐dependent, with the host playing a critical role in modulating tumor rhythms [27]. In this study, we investigate whether various immune cell populations from both the lymphoid and myeloid lineages exhibit day/night differences in distribution in healthy mice. These findings are relevant to understanding how circadian rhythms might influence immune responses and could have implications for optimizing immunotherapeutic strategies in cancer treatment. iLN samples were obtained from mice previously synchronized to a regular 12‐h light/dark (LD) cycle, sacrificed at ZT0 (light on) and ZT12 (light off), and stained with specific cell surface markers to evaluate different immune populations. Representative dot plots of the gate strategy are shown (Fig. 1).

**Fig. 2:**
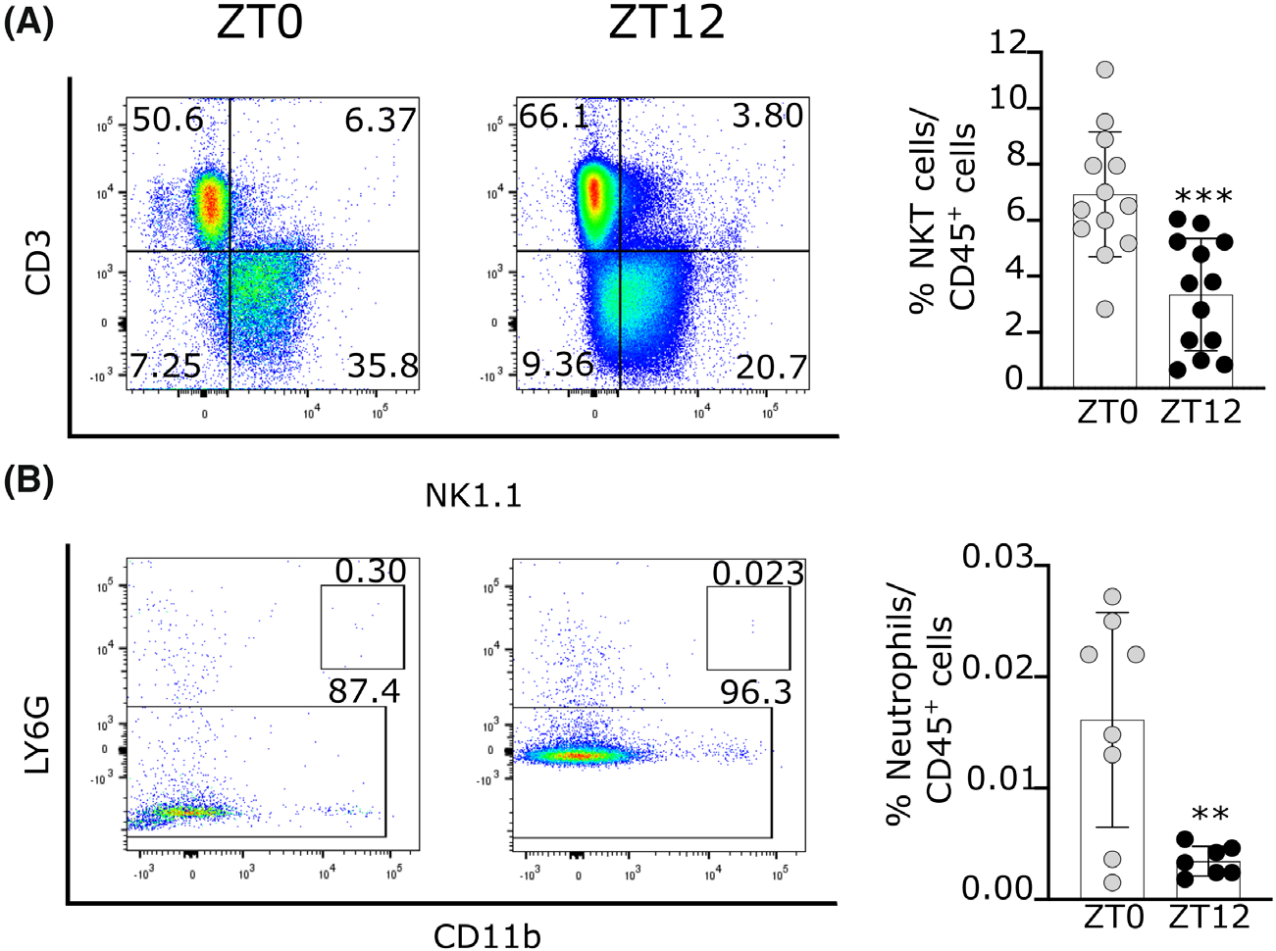
Frequency of NKT (A) and neutrophils (B) cells in iLN of mice sacrificed at ZT0 (gray circles) and ZT12 (black circles). Representative dot plots of NKT cells (CD45^+^ CD3^+^ NK1.1^+^) (*n* = 13) and neutrophils (CD45^+^ CD3^−^ NK1.1^−^) (*n* = 8 and 7 for ZT0 and ZT12 groups, respectively) according to the gating strategy. Data are the mean ± SEM of three independent experiments. **P < 0.01, ***P < 0.001 by unpaired *t*‐test.

As shown in Fig. 2, a higher proportion of NKT cells was observed in iLNs isolated from mice sacrificed at ZT0 (6.9%) compared with samples taken at ZT12 (3.3%) (*P* < 0.0002 by unpaired t‐test). Significant differences were also observed in the percentage of neutrophils, with a higher proportion observed in iLNs from mice sacrificed at ZT0 (0.016%) as compared with ZT12 (0.003%) (*P* < 0.0044 by unpaired t‐test). These observations suggested that mice showed a more permissive tumor microenvironment characterized by a reduced presence of innate immune cells at the onset of their active phase once the lights were turned off. However, dendritic, macrophage, and NK cell populations showed no significant differences between the experimental conditions evaluated (Table 1).

**Table 1:** Percentage of different immune cell populations at the iLN of mice sacrificed at ZT0 or ZT12. Data are shown as mean ± SEM of two or three independent experiments. ns, non‐significant.

Immune population		Percentage of cells (mean ± SEM)		Statistical analysis
		ZT0 (*n* = 8–13)	ZT12 (*n* = 8–13)	
Lymphoid lineage	T cells (CD3^+^)	62.3 ± 1.6	50.7 ± 4.0	ns
	CD4 T cells (CD4^+^CD3^+^)	33.5 ± 1.5	30.5 ± 1.9	ns
	CD8 T cells (CD8^+^CD3^+^)	23 ± 0.7	15.7 ± 3.4	ns
	NKT cells (CD3^+^ NK1.1^+^)	6.9 ± 0.6	3.3 ± 0.5	***
	NK cells (CD3^−^ NK1.1^+^)	10.6 ± 3.8	9.3 ± 3.2	ns
	PD1 CD4 T cells (PD1^+^ CD4^+^)	2.2 ± 0.2	1.5 ± 0.2	*
	PD1 CD8 T cells (PD1^+^ CD8^+^)	0.91 ± 0.04	0.46 ± 0.06	****
Myeloid lineage	Dendritic cells (CD3^−^ NK1.1^−^ CD11c^+^ CD11b^+^F4/80^−^)	0.16 ± 0.06	0.2 ± 0.07	ns
	Macrophages (CD3^−^ NK1.1^−^ Ly6G^−^ CD11b^+^ F4/80^+^)	0.37 ± 0.05	0.38 ± 0.2	ns
	Neutrophils (CD3^−^ NK1.1^−^ Ly6G^+^ CD11b^+^)	0.02 ± 0.003	0.001 ± 0.0005	**

*P < 0.05, **P < 0.01, ***P < 0.001, ****P < 0.0001 by unpaired *t*‐test.

**Fig. 3:**
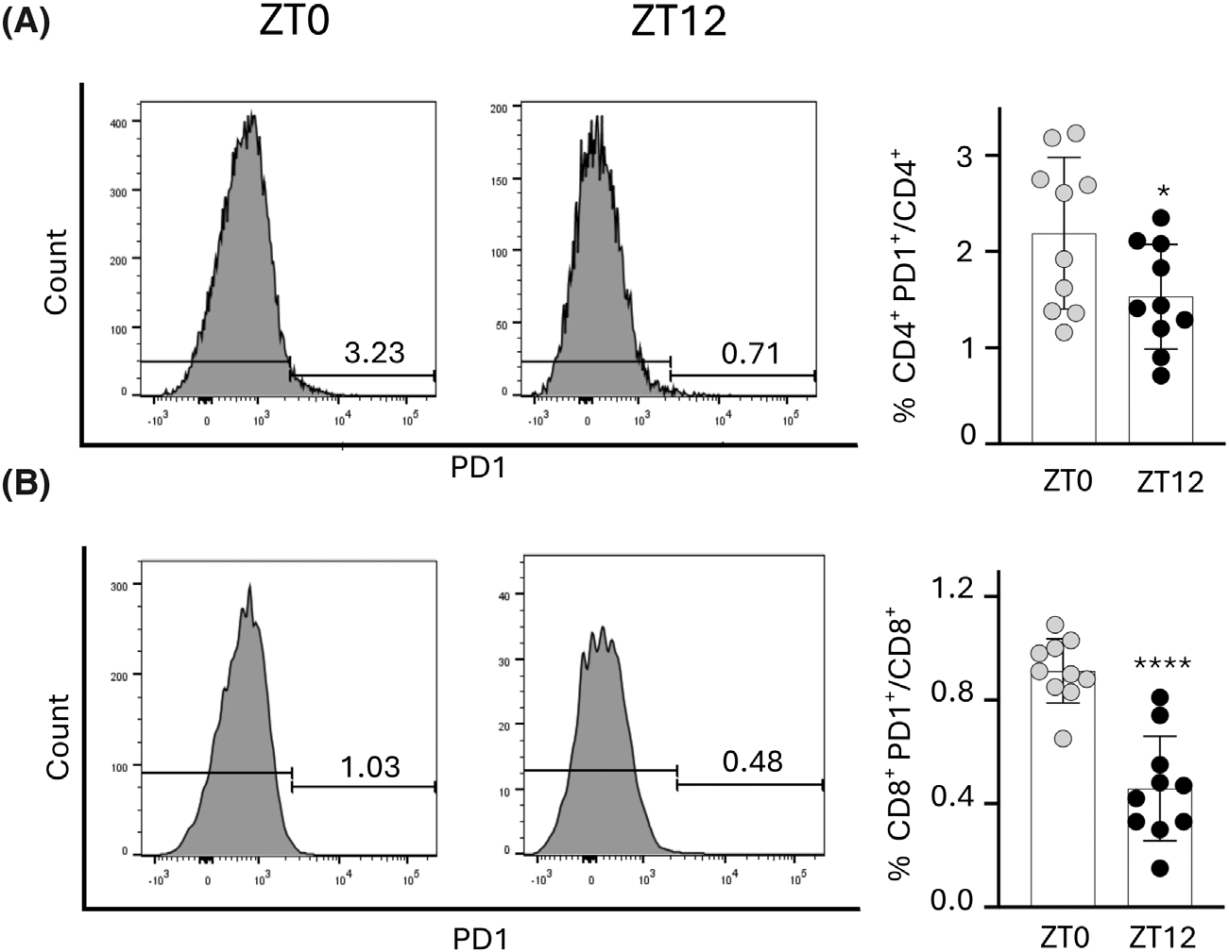
Frequency of PD1^+^ CD4^+^ T cells (A) and PD1^+^ CD8^+^ T cells (B) in the iLN of mice sacrificed at ZT0 (gray circles, *n* = 10) and ZT12 (black circles, *n* = 10). Data are shown as mean ± SEM of two independent experiments. *P < 0.05, ****P < 0.0001 by unpaired *t*‐test.

The frequency of CD4^+^ and CD8^+^ T subsets was similar in samples obtained from iLNs collected at ZT0 and ZT12 (Table 1). Remarkably, significant differences in the proportion of PD1^+^ CD4^+^ and PD1^+^ CD8^+^ T cells were found between samples of ZT0 (light on) and ZT12 (light off) (Fig. 3). Flow cytometry findings evidenced a higher frequency of PD1+ T lymphocytes PD1^+^ in the iLNs collected at the beginning of the rest phase compared to samples from mice that were sacrificed at the onset of the active phase during the early night (Fig. 3; Table 1).

## Discussion

Circadian clocks drive self‐sustained oscillations and regulate a number of physiological and behavioral processes with a period close to 24 h. Biochemical and behavioral rhythms such as the sleep–wake cycle, changes in body temperature, and hormone synthesis and release, among others, are shown to be time‐dependent. Indeed, the circadian system is involved in the temporal regulation of several aspects of the immune response, participating in defenses against various pathogens, trafficking of immune cells, activation of innate and adaptive immunity, and modulating inflammatory processes (reviewed in [30, 31, 32, 33]).

We previously reported that xenografts generated from two non‐related cancer cells showed time‐of‐the‐day differences in tumor growth rate, with significantly higher values when tumor cells were inoculated at night. Additionally, no differences were observed when tumor cells were synchronized at different times and then injected into the mice simultaneously, suggesting that the host's circadian clock plays a critical role in tumor progression [27]. To evolve and grow, tumor cells must sustain proliferative signaling, evade growth suppressors, bypass death triggers, achieve replicative immortality, encourage vasculogenesis, trigger invasion and subsequent metastasis, reprogram their metabolism, and avoid immune detection and elimination [34]. To identify which innate immune cells could mediate the difference previously observed in tumor growth rates, we evaluated the proportions of dendritic cells, macrophages, neutrophils, NKT, and NK cells in iLNs of non‐tumor‐bearing mice sacrificed at ZT0 or ZT12.

Among the different innate immune cells, neutrophils are the first line of defense, representing around 10% to 25% of circulating leukocytes [35, 36]. Like other immune cells, neutrophils express clock genes [37]. As shown in Fig. 2B, we found lower amounts of neutrophils in iLNs during the night. These findings indicate that the movement of neutrophils to lymph nodes depends on the timing, which may correlate with our earlier results demonstrating a greater tumor growth rate when nocturnally injected at the beginning of the active phase (ZT12) rather than during diurnal inoculation [27]. In cancer, neutrophils can promote or inhibit tumor growth based on varying cytokine signals [38]. In this context, a more significant number of neutrophils at the start of the rest phase (ZT0) may lead to a stronger immune response against cancer cells, which could contribute to slower tumor growth. In agreement, when neutrophils were selectively depleted at the time of *in vivo* inoculation with gamma‐irradiated tumor cells, the growth of subsequently transplanted syngeneic tumors could not be inhibited, indicating that neutrophils are important for generating specific antitumor immune responses [39, 40].

NKT cells are key players in antitumor immunity. These cells are NK1.1 + αβTCR+ cells in mice and have several characteristics that differ from conventional T and NK cells [41]. NKT cells become activated in a plethora of conditions, including infection and inflammation [42]. NKT cells are divided into two subtypes: Type I and II. Acquisition of NK receptor (NKR) NK1.1 during maturation is characteristic of the Type I subset. It is also known that Type I NKT cells are involved in the antitumor response, while their counterpart (Type II) promotes cancer progression [43]. In line with these observations, the NKT (Type I) cells present in the iLNs of mice sacrificed during the night evidence a significant reduction in their proportion compared to samples collected at the beginning of the day (Fig. 2A). Again, in this scenario, tumor cells injected during the night found a more permissive microenvironment with fewer NKT Type I cells. Interestingly, a reduced circulating NKT‐cell pool was found in cancer patients compared with healthy controls, suggesting that the size of the NKT‐cell population could have been reduced before tumor development, thereby acting as a risk factor [44].

Regarding adaptive immunity, no temporal differences in the CD8 T cell's absolute number were observed in iLN collected at opposite times (10 and 20 h). At the same time, no variations were reported in CD4 T cells from iLN harvested at these times from mice maintained under regular light/dark cycles [45]. Despite a lack of significant differences, a clear trend showed that the proportion of CD8 T cells was higher in samples collected at ZT0 compared to those taken at ZT12. Importantly, the magnitude of the response of T cells to antigen presentation by dendritic cells is shown to be controlled by the circadian machinery (reviewed in [6, 46]). It was reported a strong and efficient activation and proliferation of CD8 T cells according to the time of day. During the early stages of T cell activation, the authors observed greater T cell activation markers following vaccination in the afternoon compared to those measured at night. Naïve CD8 T cell transcriptome analysis revealed rhythmic genes associated with TCR‐dependent T cell activation, which are primed to respond more efficiently in naïve CD8 T cells in the daytime compared to nighttime [47].

PD‐1 is a cell surface receptor that is primarily expressed on T cells, B cells, and myeloid cells upon activation. It interacts with two ligands: PD‐L1 and PD‐L2, which are expressed on various cells, including some normal tissues and many types of cancer cells. When PD‐1 binds to PD‐L1 or PD‐L2, it delivers an inhibitory signal to the T cell, reducing T cell proliferation, cytokine production, and cytotoxic activity, thereby dampening the immune response. In a physiological setting, this axis is crucial for modulating the strength of immune responses and returning to homeostasis. In cancer, it is one of the most important immune checkpoints, turning T cells dysfunctional and suppressing antitumor immune responses. Thus, therapies blocking this pathway have revolutionized cancer therapy.

Evidence in the literature shows a tight crosstalk between the circadian system and the PDL‐1/PD‐1 checkpoint pathway. In fact, the clock genes RORγ, PER1, CRY2, and BMAL1 negatively regulate the expression of PD‐1 in effector T cells [48], and the expression of its ligand PD‐L1 displayed a robust circadian oscillation in normal lung tissues [49]. In melanoma‐bearing mice, Tsuruta et al. showed a diurnal oscillation in the number of PD‐1‐expressing tumor‐associated macrophages and the levels of *Pcdc1* mRNA expression [50]. Our results showed a significant difference in the proportion of PD1^+^ CD4^+^ T and PD1^+^ CD8^+^ T cells, with a higher percentage in the iLN collected from mice sacrificed at Z0 than those taken at the onset of the active phase. Interestingly, Monteiro de Assis and colleagues evidenced a positive correlation between the molecular activator BMAL1 and PD‐1 in melanoma cancer, showing that patients with high BMAL1 levels respond better to anti‐PD1 therapy than those with low BMAL1 expression [51]. Based on this evidence, we may infer that the circadian timing system may be crucial in optimizing the timing of cancer immunotherapy for improved efficacy. Additional research is required to assess PD‐1 expression in tumor‐bearing mice to explore its potential in glioblastoma immunotherapy.

Overall, our findings, together with previous reports, strongly support that the immune system is also controlled by the biological clock and that the marked day/night differences in particular immune cell subsets could condition the outcome of the immune responses. Indeed, both lymphoid‐ and myeloid‐derived cells, NKT cells and neutrophils, respectively, exhibit a higher cell proportion during the day at the inductive sites, which strongly suggests that the surveillance system could be more active in this day phase as compared to the night. In fact, the initial hours (time 0) in the generation of the immune response and the number of cells present in the lymph node are crucial for the strength of the response and will not be further compensated by a second round of the following circadian cycle [52].

Moreover, we could further speculate that a higher proportion of neutrophils at the light onset can exert more robust surveillance and defense against the appearing tumor cells or pathogenic intruders and trigger pro‐inflammatory responses against the invading cells. In addition, a higher proportion of NKT cells during the day in the infiltrating area could promote the release of interferon‐gamma to attack newcomers.

Our results, presented herein, highlight the pivotal role of characterizing circadian clock modulation under basal conditions in the mouse, establishing a foundation for the rational design of strategies aimed at eradicating tumor cells with maximal efficiency. By providing a comprehensive understanding of how the immune system is regulated in relation to the biological clock, our study unveils a powerful and innovative framework that can drive the development of next‐generation antitumor therapies. Further studies will be needed to elucidate the precise connections between the day/night changes in immunity, the circadian system, the disease progression, and chemotherapeutic treatment.

## Conflict of interest

The authors declare no conflict of interest.

## Author contributions

All authors contributed to the study conception and design. Material preparation and data collection were performed by PMW, CGP, LB, and VAP. MEG, SGC, and MM contributed the reagents and analytic tools. All authors analyzed data and results. The first draft of the manuscript was written by PMW, CGP, and MEG, and all authors commented on previous versions of the manuscript. All authors read and approved the final manuscript.

## Supporting information


**Table S1.** Antibodies used for flow cytometry analysis.

## Data Availability

Data will be made available on request.
