# Supplementary material for: Day/night variations of myeloid and lymphoid cell subsets in the murine inguinal lymph node
Source: FEBS Open Bio. 2025 Nov 5;16(3):487–95. doi: 10.1002/2211-5463.70137 (PMC12955754; doi:10.1002/2211-5463.70137)
Supplement: Supplementary file 1 — Table S1. Antibodies used for flow cytometry analysis. [file FEB4-16-487-s001.docx]

**Suppl. Table 1.** Antibodies used for flow cytometry analysis

| **REAGENT** | **FLUOROCHROME** | **SOURCE** | **IDENTIFIER/CLONE** |
| --- | --- | --- | --- |
| LIVE/DEAD | BV510 | Invitrogen | #2445379 |
| CD45 | APCCY7 | Biolegend | #103116/30-F11 |
| CD3 | PECY7 | Invitrogen (eBioscience) | #2460205/145-2C11 |
| CD4 | AF700 | Biolegend | #100429/GK1.5 |
| CD8 | FITC | BD | #553030/53-6.7 |
| CD11b | BV711 | Biolegend | #101241/M1/70 |
| LY6G | PE | Biolegend | #127607/1A8 |
| NK1.1 | PECY5 | Biolegend | #108715/PK136 |
| PD1 | BV786 | Biolegend | #135225/29F.1A12 |
| CD11c | PECF594 | Biolegend | #117347/N418 |
| F4/80 | AF647 | Biolegend | #123122/BM8 |
